# Femoral neck non-union treatment by valgus intertrochanteric osteotomy

**DOI:** 10.1590/1413-785220152306148750

**Published:** 2015

**Authors:** Carlos Roberto Schwartsmann, Leandro de Freitas Spinelli, Anthony Kerbes Yépez, Leonardo Carbonera Boschin, Marcelo Faria Silva

**Affiliations:** 1 Universidade Federal de Ciências da Saúde de Porto Alegre, Santa Casa de Misericórdia de Porto Alegre, Ortopedia e Traumatologia, Porto Alegre, RS, Brasil; 2 Santa Casa de Misericórdia de Porto Alegre, Departamento de Ortopedia e Traumatologia, Cirurgia do Quadril, Porto Alegre, RS, Brazil; 3 Universidade Federal de Ciências da Saúde de Porto Alegre, Fisioterapia, Porto Alegre, RS, Brasil

**Keywords:** Femoral neck fractures, Osteotomy, Hip/surgery.

## Abstract

**OBJECTIVE:**

: The purpose of this study was to evaluate the performance of valgus intertrochanteric osteotomy in femoral neck non-union.

**METHODS:**

: Forty-two patients with femoral neck fractures with non-union treated using Pauwels' intertrochanteric osteotomy were reviewed. Demographics, time elapsed between fracture and surgery, follow--up, osteosynthesis used, Garden's classification, limb shortening, and x-rays were evaluated.

**RESULTS:**

: Twenty-two men and 20 women were reviewed. The youngest patient was 18 years old and the oldest 65 years old, with a mean age of 42.4 years (±11.2). The minimum follow-up was 2 years, with a mean of 10.2 years. The average time elapsed between initial fracture and osteotomy was 6.5 months. Twel-ve cases were neglected femoral neck fractures. Nineteen patients were classified as Garden III, and 23 patients as Garden IV. After valgus osteotomy, non-union healing was observed in 38 patients (38/42; 90.4%). Healing of thirty-seven cases of pseudoarthrosis were obtained after the first-attempt osteotomy, and one case required two operations for healing. The osteotomy failed in four cases. Conside-ring the healed osteotomies, good to excellent functional results were achieved in 80.9% (34/42) of the patients. Total hip replacement was subsequently performed in 14.2% (6/42) of the patients for unfavoura-ble outcomes (two for cutting out, two for osteonecrosis, and two for osteoarthritis).

**CONCLUSIONS:**

: Valgus intertrochanteric osteotomy has a high success rate in archiving healing in femoral neck non-union with good functional results. It is a biological and effective method. **Level of Evidence IV, Therapeutic Study.**

## INTRODUCTION

Non-union and avascular necrosis of the femoral head are the main complications of fractures of the femoral neck. The high rate of occurrence of such complications is due to a combination of unfavourable biomechanical and vascular conditions caused by the fracture itself. Several non-union treatments were described and include arthroplasties, osteotomies with or without bone graft, and different techniques of vascularised bone grafting.

Pauwels showed that a higher shearing angle could lead to an unfavourable consolidating process. For this reason, a valgus osteotomy converts shearing into compression forces and increases the fracture healing potential.^1^ The consolidation of the pseudoarthrosis with the preservation of the femoral head is the biological alternative and can offer the best long-term results for the young patient. However, if the patient is older than 65 years and presents a displaced fracture, there is a consensus for the use total hip replacement, eliminating the recurrence of the main complications of the biological alternative (pseudoarthrosis and aseptic necrosis) and providing rapid pain relief and early mobilization.

The purpose of this study was to evaluate the performance of valgus intertrochanteric osteotomy in the treatment of femoral neck non-union.

## METHODS

We retrospectively reviewed 42 patients who had undergone valgus intertrochanteric osteotomy from 1990 to 2011 in our hos-pital. Demographics, time elapsed between fracture and surgery, follow-up, osteosynthesis used, Garden's classification, limb shortening, and x-rays were evaluated. The study was approved by the Institutional Ethics Committee, protocol 12/991.

The decision to treat with valgus intertrochanteric osteotomy was based on chronological and physiological age (less than 65 ye-ars), good bone stock, and sphericity of the femoral head based on the x-rays obtained. Osteoporotic bone, presence of osteo-necrosis or articular incongruence or excavation of the femoral head were considered as a contraindication to the procedure. The surgical technique adopted was valgus intertrochanteric osteotomy. We performed surgery using the lateral approach with the patient on an orthopaedic table and an image intensifier. After split vastus lateralis and removing any existing implant, a guide pin helped us find the appropriate angle for inserting the new hardware. An oscillating saw was used and a pre-determined wedge was performed. The angle of the wedge depends on the inclination of the non-union line. The average wedge angle was 32^o^. The osteotomy was performed in lesser trochanter level with complete wedge resection. Cancellous bone from the wedge was apositioned around the osteotomy site. Twenty-seven osteotomies were fixed with 130^o^ DHS, ten with a 130^o^ fixed angle blade-plate, and five with the same plate plus rotatory screw.

Patients were permitted to ambulate with partial weight bearing only 6 weeks after surgery and full weight bearing was encou-raged after 12 weeks. The verification of the consolidation process of the non-union, and either the osteotomy, was per-formed by means of x-rays. Functional analysis was performed by the Harris Hip Score, and osteonecrosis was evaluated by the Ficat's classification. Discrepancies were measured by tape measure. Mechanical modifications induced by the osteotomy were evaluated and measured. A comparison with the contralateral (normal) side was also performed.

## RESULTS

Twenty-two men and 20 women were included in the study. The youngest patient was 18 years old and the oldest was 65 years old; the average age was 42.4 years (±11.2). The minimum follow-up was 2 years, with a mean of 10.2 years, ranging from 2 to 21 years.

The average time elapsed between initial fracture and osteo-tomy was 6.5 months, ranging from 3.5 to 12 months. Twelve cases were neglected femoral neck fractures.

Thirty patients had osteosynthesis as the initial treatment. For first treatment, the majority were fixed with three cannulated screws (19 cases). Seven patients were fixed with DHS, three with a 130^o^ fixed angle blade-plate and one with three Knowles wires. According to Garden's classification, 19 patients were classified as Garden III, and 23 patients as Garden IV.

**Figure 1:**
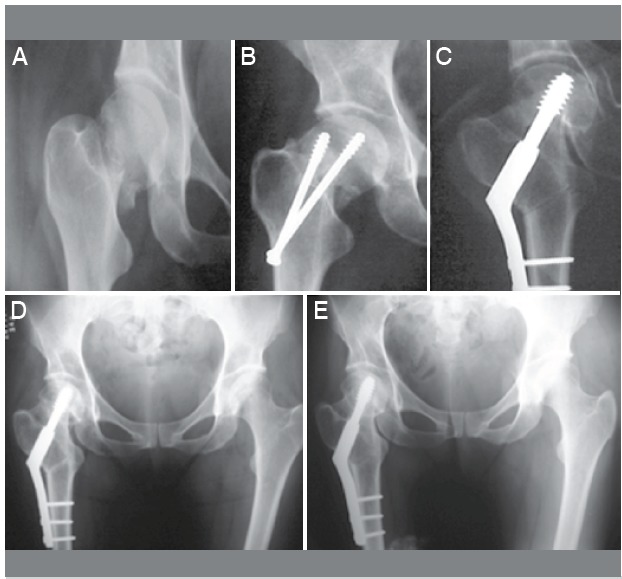
Femoral neck fracture (A); (B) failure of initial osteosynthesis treat-ment with two cannulated screws; (C) post-operative valgus intertrochanteric osteotomy; (D) and (E) pelvis x-ray showing the consolidation of valgus os-teotomy and healing of non-union neck fracture in one year and 7 years later.

Non-union healing was confirmed radiographically and was achieved in 38 patients (90.4%) with good functional outco-mes (Figures 1 and ^2^). Thirty-seven pseudoarthrosis healings were obtained after first-attempt osteotomy. Non-union at the fracture site persisted in one patient. We achieved consolida-tion after revision osteotomy involving changing the hardware device (130^o^ fixed angle blade-plate changed to DHS).

The osteotomy failed in four cases. All of these patients had undergone total hip replacement. In two patients failure was due to the hardware cutting out the femoral head and in ano-ther two the femoral head collapsed due to osteonecrosis during the weight-bearing period.

The 38 healing non-union were analysed according to Ficat's classification for osteonecrosis using plain radiographs.^2^ We considered 31 femoral heads to be normal. Six were classified as Ficat stage 2. Another case developed to stage Ficat 3 at 6 years. After removing the hardware (DHS) and bone graf-ting, the necrosis stabilized. After 14 years of follow-up of this case, total hip replacement was not considered necessary. Two patients developed osteoarthrosis, and arthroplasty was performed at 5 and 8 years post-operatively. The average Harris Hip Score^3^ obtained in the last visit for the 36 remaining patients was 81.2 points (±7.2), ranging from 74 to 96.

In the 24 patients with limb shortening pre-operatively, limb length equalization was achieved in 16 cases (16/24). The ave-rage limb shortening was 2.5 cm, ranging from 1.0 to 3.2 cm. The average abductor length was 64.8 mm (54-75 mm) for the osteotomy side. The contralateral (normal) side was 73.7 mm (63-92 mm). Therefore, there was an average reduction of 8.9 mm. Percentage-wise, there was a decrease of 12.1% in the abductor length.

The average neck shaft angle for the normal side was 132º (120-145º). The average angle after osteotomy was 144º (131-152º). Therefore, there was a valgization of 12^o^. The per-centage valgization compared to the normal side was 9.2%. None of the patients had complained about pain or deformity in the homolateral knee.

## DISCUSSION

Femoral neck fractures have been described in the past as "the unsolved fracture".^4^ Despite improved operative techni-ques, surgical technologies, hardware material and theoretical understanding, osteonecrosis and non-union continue to be the main complications of fracture of the femoral neck.

Non-union rates of 10% to 59% have been reported and may be seen more frequently in young patients due high-energy trau-ma.^5^ ^14^ In this series, the average age was 42.4 years (±11.2). The aetiology of non-union is usually unknown and multifac-torial. Several studies have attempted to describe predictive characteristics for the fracture in order to describe its evolution to pseudoarthrosis.^9^ ^15^ ^19^ Fracture displacement appears to be the most reliable predictor. Posterior comminution, fracture level, delayed surgery, inadequate reduction and poor internal fixation have also been reported.

**Figure 2:**
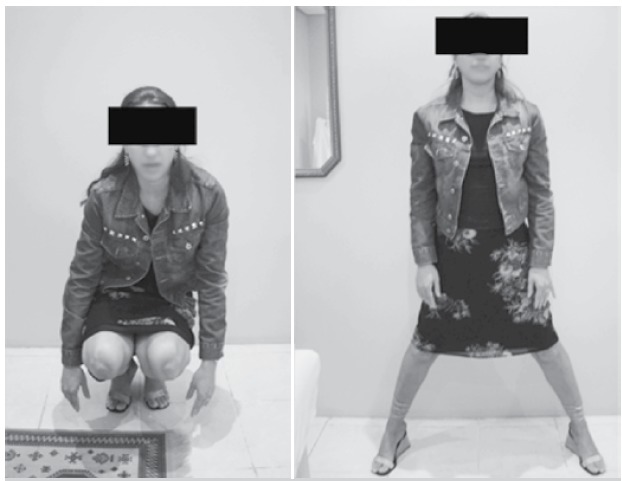
Functional results after healing of neck non-union.

To date there are no established criteria for the diagnosis of non-union of femoral neck fractures. Most authors have concluded that the definitive diagnosis should be based on time (more than 6 months), deterioration in patient's function, and worsening of pain in groin or buttock, aggravated by weight bearing or under rotation. After clinical history, serial radiographs are the most reliable parameter for consideration. Changes in screw position, change in fracture position, rea-bsorption of fracture baseline, and backing out of screws are some of the criteria that should be considered.^5^ ^6^ ^9^ ^10^ ^16^

In a few recent cases, MRI, CT scans, and isotope bone scans were used for the diagnosis of necrosis, but x-rays were the most important criterion used in image examination. Radio-graphic avascular necrosis without collapse is not an absolute contraindication in preserving the femoral head. Acceptable results have been reported in the absence of collapse.^6^ ^20^ ^21^ Osteonecrosis with incongruence, poor quality bone and rea-bsorption, disruption or excavation were considered as a con-traindication for osteotomy. In our series, the average interval between injury and valgus osteotomy was 6.5 months. Although most authors fix the osteotomy with a 130^o^ fixed angle blade--plate we used the DHS 135º in 64.3% of cases (27/42). We prefer the dynamic hip screw rather than a blade plate. The dy-namic hip screw provides rigid stability, is less aggressive, allo-ws compression in non-union fracture and is easier to perform. Bone healing in non-union was achieved in 38 cases (90.4%). Cases that failed to achieve bone healing were associated with inappropriate surgical technique. In two cases the dyna-mic screw cut out the femoral head and another two cases were associated with collapse and necrosis of the head. In one case the introduction and length of the blade of the plate were inappropriate and in another, the blade was too long and cau-sed distraction and necrosis during the weight-bearing period. All 42 trochanteric osteotomies achieved complete healing. We achieved healing in 41 cases (97.6%) at the first attempt. Revision osteotomy was performed in one patient. After chan-ging the hardware (Blade plate for dynamic hip screw) and bone grafting, healing was also achieved in this patient.

 Many papers agree that Pauwels' intertrochanteric osteotomy is a reliable option for achieving healing in non-union femoral neck fractures. Ghosh et al.^21^ reported a consolidation rate of 86% (30/35), Pidhorz et al.^22^ 74%, Marti et al.^6^ 86% (43/50), Mathews and Cabanela^23^ 80% (12/15), Lies and Scheuer^24^ 88% (15/17), Eid^25^ 90% (9/10), Ballmer et al.^7^ 88% (15/17), Raaymmakers and Marti^26^ 88% (58/66), Wu et al.^27^ 94% (16/17), Magu et al.^28^ 93% (14/15), and Zehi et al.^20^ 98% (40/41). Many authors re-ported 100% of non-union healing. Anglen^29^ reported 13 cases, Wentzensen and Weller^30^ 7 cases, Walcher and Wiesinger^31^ 13 cases, Varshney and Trikha^32^ 7 cases, and Min et al.^33^ 11 cases. Despite the high consolidation rate in many cases, osteone-crosis with or without collapse may occur. Clinically good re-sults have been associated with this situation.^6^ ^7^ ^20^ ^26^ However, if degenerative changes progress, total hip replacement could become necessary. Obviously, as the duration of follow-up increases, the number of hip replacements increases. Marti et al.,^7^ in 7 years follow-up, reported 14% of hip replacements, Mathews and Cabanela^23^ reported 16% after 4 years, Ballmer et al.^7^ 12% after 3 years, Wu et al. 27 12% after 2 years, and Min et al.^33^ 18% after 5 years (2/11). In our series, we recorded 14% (6/42) after 10.2 years.

Mathews and Cabanela^23^ were the first to measure anatomical and biomechanical alterations after valgus intertrochanteric osteotomy. After reviewing 15 cases, the authors concluded that abductor moment decreased, on average, by 11 mm when compared with the normal opposite side. In the present study we found a reduction of 8.9 mm. Theoretically, the ab-ductor moment decreased by an average of 12.1%.

Mathews and Cabanela^23^ also analysed the neck shaft angle. In comparison with the opposite side, the angle increased from 123º to 149º. In our cases, the average increased from 132º to 144º. The main criticism of this alteration is the ne-gative influence of pelvitrochanteric muscular strength and the repercussions in the homolateral knee. For this reason Matheus attributed limping in 96.6% of cases (13/15). On other hand Ballmer et al.^7^ only found 15.4% (2/15) of limping. In the present study, at least during the follow-up period, mild Trendelenburg gait was observed in five cases (5/36 - 13.2%). None of the patients used a stick or crutches.

Limb shortening was observed in 24 patients. Valgus trochan-teric osteotomy has the potential to correct leg length discre pancies. Restoration of leg length can be achieved by valgization and varying the size of the wedge taken from the distal fragment. During the follow-up period, the average shor-tening in 8 patients was 12 mm. Equalization was achieved in 16 cases (16/24). This means that 66.6% of the discrepancies were corrected.

After excluding complicated cases, 36 of the remaining os-teotomies showed good functional outcome as evaluated by the Harris hip score. The average hip score was 81.2 points (±4.2). Hip function was excellent in 14 patients (14/36). Good results were obtained in 20 (20/36) and fair in 2 (2/36). Good and excellent functional results were reported by many 6,7,20,23,26,27,29,30,33

Another alternative for non-union could be total hip arthroplasty (THA), which can offer very good outcomes. Unlike osteotomy, THA provides rapid pain relief and allows early mobilization. However the long-term results of hip arthroplasties are not always as expected.^41^ ^45^ High failures rates and bad results have been reported in young patients. In such patients, total hip replacement could be an easier solution, but probably not the best. Valgus trochanteric osteotomy is a less radical approach and is worth considering.

## CONCLUSIONS

Valgus intertrochanteric osteotomy achieved 90.4% of hea-ling in the treatment of femoral neck non-union (38/42). It is a biological and effective method for the treatment of neck non-union with good functional results. 
